# Workplace Harassment of Transgender People: A Narrative Review

**DOI:** 10.3390/bs16040479

**Published:** 2026-03-24

**Authors:** RJ Kubicki, Joseph A. Vandello

**Affiliations:** Department of Psychology, University of South Florida, Tampa, FL 33620, USA; rkubicki@usf.edu

**Keywords:** transgender employees, gender identity, employment discrimination, workplace harassment, LGBTQ+ employees

## Abstract

Workplace harassment of transgender employees remains pervasive and understudied. In this narrative review of 63 studies over the past 25 years, we summarize the literature on transgender workplace harassment. We focus on its prevalence and forms. Individual, organizational and cultural factors contribute to its occurrence; psychological and occupational outcomes; and strategies to reduce or prevent harassment. We find that harassment often extends beyond traditional definitions; includes misgendering, deadnaming, and the questioning or outright denial of one’s gender identity; and is particularly pervasive in masculinity contest cultures. These experiences are associated with both negative well-being of transgender employees and less effectiveness of the organizations that employ them, though more causal evidence is needed. We also highlight critical conceptual and methodological gaps to guide future research. Much of the existing research on LGBTQ+ employees in the workplace has focused primarily on sexual minorities, leaving the unique experiences of gender minorities invisible. Further, an intersectional lens is needed, as harassment experiences of trans women, trans men, and nonbinary people may differ in significant ways. Finally, we identify strategies to improve workplace climate including both top-down formal policy and bottom-up interpersonal behaviors.

## 1. Introduction

With the increased visibility of transgender people in recent years, driven by greater media representation; social activism; and fluctuating, often volatile, legal recognition, comes the possibility of increased vulnerability to workplace harassment. Yet, organizational responses to workplace harassment targeting transgender people often lag behind these social shifts (Vargas et al., 2022). Traditional harassment policies were largely written from heteronormative perspectives, with cisgender, heterosexual men and women in mind, and therefore may fail to account for the experiences and needs of transgender populations. The goal of this paper is to review the past 25 years of research on the workplace harassment experiences of transgender people through the lens of cisnormativity. The workplace is a public space where gender norms are reproduced, reinforced, and policed. Through this lens, we hope to show how transgender harassment is both pervasive and underappreciated, as some forms of harassment may go unrecognized (both legally and by lay persons). A cisnormative lens exposes current limitations and blind spots in the current scientific understanding of workplace harassment, as most theoretical understandings of harassment (and workplace policies) were formed with cisnormative assumptions that may be inadequate to account for the unique experiences of trans workers. Similarly, cisnormative assumptions have also limited the empirical literature on transgender harassment by reproducing gender binaries that render nonbinary people largely invisible.

Workplaces are not neutral spaces; rather they are structured by cisnormative assumptions that can create gender hierarchies, segregation, and exclusion (Öztürk, 2024; Worthen, 2016). Cisnormativity means that cisgender people (people whose gender identity corresponds to their sex assigned at birth) are the default, and they are privileged over trans and nonbinary people. This does not always happen consciously or intentionally, but can be conveyed in language, expectations, and the structure of the workplace (Ericsson, 2018). Cisnormative organizational structures and cultures (Köllen & Rumens, 2022) can license prejudice and hostility towards trans people for being “not normal”, yet not all negative workplaces meet the threshold of harassment. In addition, some forms of harassment may be largely invisible to all but transgender people– exclusion and microaggressions, for example.

Scholarly understanding of workplace sexual harassment has shifted substantially over the past 50 years. From the time the term sexual harassment entered public and academic discourse in the mid-1970s, scholars conceptualized harassment as an expression of sex-based power, not sexual desire (Fitzgerald, 1990). For example, MacKinnon’s (1979) influential definition stated that harassment is “the unwanted imposition of sexual requirements in the context of a relationship of unequal power” (p. 1). By the 1980s, U.S. courts recognized two major forms of harassment: Quid pro quo harassment (benefits tied to compliance) or a hostile work environment (conduct making work intimidating or unpleasant) (Dickinson, 1995). But these early efforts emphasized overt sexual advances over subtler behaviors and often assumed an implicit cis-male harasser/cis-female victim dyad. By the early 2000s, harassment scholars began using the term gender harassment to distinguish from sexual coercion or attention. Harassment was reconceptualized as a mechanism for maintaining gender hierarchies, not merely pursuing sex (Berdahl, 2007; Fitzgerald & Cortina, 2018).

Harassment may occur at both interpersonal and structural levels, reflecting broader organizational and societal norms that privilege cisgender identities. As we will show in this review, some aversive behaviors commonly experienced by transgender employees (e.g., microaggressions, intrusions, and exclusions) may go unrecognized as gender harassment under current psychological and legal definitions, but are certainly experienced as hostile work environments.

We propose that transgender harassment can be best understood from the perspective that much harassment stems from a motivation to enforce gender norms when they are perceived to be violated (Berdahl, 2007). Role congruity theory (Eagly & Karau, 2002) argues that those perceived as gender deviants (and transgender people certainly are perceived by some as deviants and transgressives) are targeted for punishment as a way of enforcing gender norms that spring from cisheteronormative assumptions. We further propose that harassment is especially likely in environments invested in maintaining male-dominated gender hierarchies. Some workplaces have been characterized as “masculinity contest cultures” where men feel pressure to prove themselves as “real men” leading to dysfunctional climates conducive to bullying and harassment (Berdahl et al., 2018). Thus, it is likely that transgender people are more vulnerable to harassment in some environments than others.

The aim of this paper is to review the literature on the prevalence, forms, causes, and outcomes of transgender workplace harassment. More specifically, four questions guided our narrative review (1) How prevalent is workplace harassment for transgender employees, and what forms does it take?; (2) What individual, organizational, and cultural factors contribute to its occurrence?; (3) What psychological and occupational outcomes result from these experiences?; and (4) What strategies, ranging from legal protections to cultural change initiatives, can reduce or prevent harassment? By reviewing findings from the past 25 years of research, these questions seek to deepen theoretical understanding of gender-based harassment and guide the development of effective interventions that foster a safer and more equitable workplace for transgender employees. We end by suggesting avenues for future research.

## 2. Methods

We conducted a narrative review of research on the workplace harassment of transgender people informed by a systematic search of relevant literature. Our search was guided by the four questions above in order to get a picture of the frequency and forms of harassment that transgender workers experience, the correlated with their mental health and work outcomes, and the strategies that might improve workplace experiences of trans workers. Relevant articles were identified through searches in four databases in April, 2025: Ebsco, PsycInfo, Scopus, and Web of Science. The search was limited to studies published in or after the year 2000, as most empirical research on transgender populations has emerged since that time (interestingly, the earliest study we found on the topic was from 2009). Boolean search terms included combinations of “transgender”, “LGBTQ”, “workplace”, “work”, “employee”, “organization”, and “harassment”. These searches yielded 1491 unique articles. Titles were first screened by the first author to exclude studies unrelated to workplace settings (e.g., school or university environments), non-empirical papers (though we did include non-peer-reviewed agency reports that included primary data), unpublished theses, non-English publications, and studies that did not focus on harassment. For example, some studies did not include transgender respondents; other studies surveyed transgender respondents about workplace attitudes but did not include harassment experiences. Both authors reviewed abstracts of the remaining studies to confirm relevance. Relevant articles were coded for the inclusion of content addressing one or more of the main research questions (concerning harassment frequency, forms, individual and organizational contributors, mental health and well-being outcomes, and policies and strategies to mitigate harassment). Additional articles were identified through citation chaining of papers found in the initial search, leaving 64 empirical papers. In addition to these papers, we also supplemented this review with relevant research from broader literature on workplace harassment and transgender prejudice when and where appropriate. Rather than providing an exhaustive summary, we aimed to synthesize overarching themes and identify conceptual and methodological gaps in understanding the workplace experiences of transgender employees.

## 3. Results and Discussion

### 3.1. Frequency and Forms of Harassment

Accurately estimating the prevalence of workplace harassment faced by transgender people is challenging. Few representative surveys exist, and those that do often combine transgender individuals with other LGB+ groups, use “LGBTQ+” without considering gender minorities’ unique experiences or fail to distinguish harassment from discrimination. Furthermore, studies vary with respect to sampling methods, workplace context, culture, time periods assessed (some report past year experiences, others, lifetime), and heterogeneity in definitions of harassment, leads to a wide range of estimates. Nevertheless, available research suggests that harassment is both widespread and, in some contexts, a normative workplace experience.

Table 1 summarizes the 15 studies we located reporting the prevalence of transgender workplace harassment. Within the U.S., there have been at least four large surveys of transgender employees. The first, the 2008–2009 National Transgender Discrimination Survey which included 4597 transgender and nonbinary respondents, showed that half reported having been harassed at work, with 7% reporting physical violence and 6% sexual assault due to their gender identity (Enogieru et al., 2024), A second large survey of over 27,000 transgender adults, the 2015 U.S. Transgender Survey, found that 15% were verbally harassed, physically attacked, and/or sexually assaulted at work in the past year alone; 23% reported other forms of harassment, such as being told by their employer to present as the wrong gender in order to keep their job or having coworkers disclose private information about their gender identity (James et al., 2016). More recent data from the Center for American Progress (Medina & Mahowald, 2023) surveyed over 1800 LBTQI+ adults. Among transgender respondents, 70% reported experiencing some form of workplace harassment or discrimination in the past year: 51% reported verbal harassment, 41% reported sexual harassment; 21% physical harassment; 26% were excluded from work events; and 23% were denied access to restrooms. Finally, a national survey of LGBTQ+ American workers found that among 84 transgender or nonbinary participants, 68% had experienced at least one form of workplace harassment (verbal, physical, sexual) during their careers, and 56% had experienced harassment in the past five years (Sears et al., 2024). Transgender employees were also significantly more likely than cisgender LGBQ+ employees to report harassment. It is worth noting that these most recent surveys reported higher rates of harassment than the earlier surveys. While we caution against making direct comparisons across surveys, it is certainly plausible that harassment experiences have become more common in the past ten years as transgender issues have become more prominent and polarizing.

Harassment also appears especially pronounced in many professional settings, particularly in work environments having masculinity contest cultures (Berdahl et al., 2018). For example, an overwhelming 83.9% of transgender service members in the U.S. military reported experiencing sexual harassment (Schuyler et al., 2020). Patterns are similarly troubling in scientific fields. In a survey of 324 U.S. LGBT physicists, nearly half (49%) of transgender respondents reporting “exclusionary behavior” (i.e., being harassed, shunned, or ignored) in the past year, greater than the 22% of LGBT respondents in total (Barthelemy et al., 2022). This rate was similar to another survey of 1005 LGBTQ+ professionals across 21 STEM disciplines, in which about 20% reported experiencing harassment in the past year (Cech & Waidzunas, 2021). These patterns extend to healthcare, with a great deal of research finding similar patterns. This indicates that even ostensibly inclusive environments may foster subtle, yet pervasive forms of bias and harassment (Cook et al., 2025; Chester et al., 2014; Jongbloed et al., 2025).

Outside of the U.S., several studies have probed the workplace harassment experiences of transgender employees. A study of 235 Canadian Federal Public service workers found that 31% reported harassment in the past two years (Waite, 2021). A large European Union LGBTIQ Survey from 2023 found that a majority of trans people in every country queried reported being harassed in the past 12 months, but this was not specific to the workplace (European Union Agency for Fundamental Rights, 2024). An Australian national online survey of 1000 LGBTQ young people aged 14–30 found that 77% of experienced workplace sexual harassment (transgender respondents were not separated from other LGBTQ groups; Robinson et al., 2024). A Chinese survey of over 10,000 LGBTI workers included 691 trans people; 31.4% reported “negative treatment” (higher than the 21.2% overall in sample; Suen et al., 2021). An Italian study of 72 transgender people found that 22% had ever experienced harassment or discrimination at work (Prunas et al., 2018).

Thus, across work environment and cultures, harassment experiences are quite common, and even more common than among cisgender LGBQ+ populations (for whom harassment is also common) or cisgender heterosexual populations. It is also worth noting that despite facing disproportionately high rates of sexual harassment, transgender employees may be more likely to have their experiences of harassment ignored or questioned by others (Bandt-Law et al., 2025). It is important to be cautious in comparing rates across these studies because some studies reported rates for transgender respondents separately from other LGBTQ+ groups, while other studies grouped them together. Some studies ask about harassment specifically within the workplace, while others ask about harassment more generally. Studies also vary in the time range asked about (e.g., ever versus in the past year). Table 2 summarizes ten studies that report the types of behaviors that comprise the workplace harassment of transgender people. The forms that workplace harassment takes for transgender people often mirror those faced by other marginalized groups, and includes verbal abuse, bullying, name-calling, hostile jokes, and sexual harassment (Brewster et al., 2014; Waite, 2021; Watson et al., 2019). However, transgender employees also face unique forms of harassment rooted in cisnormativity (Brewster et al., 2012). These include restroom surveillance and exclusion (Herman, 2013), misgendering and deadnaming (Perales et al., 2022; Resnick & Galupo, 2019; Van de Cauter et al., 2021), and subtle, often unintentional microaggressions (Cancela et al., 2024). Examples include invasive questioning about one’s transition (Baiocco et al., 2023), scrutiny of competence (Van de Cauter et al., 2021), criticism of gender expression (Galupo & Resnick, 2016), or being told to “tone it down” to avoid making others uncomfortable. Because microaggression are frequently ambiguous or passive-aggressive, they often go unrecognized in formal organizational settings, leaving them unaddressed. Current workplace anti-harassment policies rarely capture the distinct and context-specific forms of harassment experienced by transgender employees, perpetuating organizational blind spots and leaving employees further vulnerable to harm. This research suggests that current definitions of harassment (both legal and scientific) need to be expanded to include trans-specific experiences.

### 3.2. Interpersonal, Organizational, and Cultural Contributors of Harassment

Research on individual-level predictors of the harassment that transgender people experience in the workplace remains limited. However, studies on predictors of prejudice toward transgender people, as well as studies of harassment toward women and LGB employees, can offer important insights. For example, transphobia has been found to correlate strongly with right-wing authoritarianism, religious fundamentalism, political conservativism, traditional gender role beliefs, and hostile sexism (Hatch et al., 2022; Nagoshi et al., 2008; Wang-Jones et al., 2017), supporting the perspective that harassment is often motivated by threats to those holding traditional gender role beliefs and targeted at those perceived as “gender deviants.” Similarly, men, particularly those whose masculinity feels threatened (Berdahl, 2007; Maass et al., 2003), individuals high in hostile sexism (Bareket & Fiske, 2023), and people with conservative ideologies (Ortiz-Bonnin & Blahopoulou, 2025) are more likely to sexually harass women or gender nonconforming coworkers, or to endorse harassment-tolerant attitudes. Pre-existing prejudice at the individual level can distort perceptions of harassment toward transgender people, contributing to hostile work environments and the normalization of mistreatment. Even when harassment occurs, it may be perceived differently depending on the target’s identity. Brassel et al. (2019) found that lay observers viewed harassment directed at transgender employees as less severe and less clearly motivated by bias than similar behaviors targeting lesbian or gay employees. These perceptual biases can lead to the minimization of harassment experiences and weaker institutional responses, further reinforcing environments where the mistreatment of transgender workers is overlooked or dismissed.

Johnson et al. (2022) examined the role of identification strength with one’s transgender identity as a factor in workplace mistreatment, hypothesizing that employees who strongly identify with their transgender identity would face higher rates of mistreatment following harassment. For cisgender individuals with high levels of prejudice, this was indeed the case. However, they also observed a favorability bias among cisgender participants low in prejudice, such that strongly identified transgender employees received greater support and significantly lower termination endorsement. This pattern suggests that transgender employees who are highly identified with their identity may align more closely with organizational prototypes of inclusion, resulting in a positivity bias from less prejudiced or diversity-trained individuals, while those less overtly identified receive less support and recognition. Eagly and Karau (2002) proposed in their Role Congruity Theory that people in the workplace who lack congruence with traditional gender role expectations will be most likely to face negative consequences. In other words, people face negative evaluations when their gender presentation conflicts with stereotypical gender expectations. Although this framework was originally applied to cisgender women, it is highly relevant to transgender employees. For example, Currin et al. (2022) found that adherence to traditional gender roles and masculine self-presentation predicted negative attitudes towards transgender employees. Similarly, Rice et al. (2022) found that transgender employees were often rated as less competent than their equally qualified cisgender coworkers. These findings suggest that role incongruence may contribute to workplace harassment and biased evaluations of transgender employees. Moreover, evidence indicates that transgender women may be particularly vulnerable to sexual harassment in the workplace, reflecting broader gender hierarchies (Schilt & Wiswall, 2008; Suárez et al., 2022). Women in general, regardless of sexual orientation, report higher rates of workplace sexual harassment than men (Jongbloed et al., 2025), and this disparity may extend to transgender women (Devis-Devis et al., 2022), who occupy both marginalized gender and gender-identity positions. Research also suggests that others may perceive harassment toward transgender women as less severe or less deserving of intervention than harassment toward cisgender women, further minimizing their experiences and compounding their vulnerability (Mezzapelle & Reiman, 2022). Consequently, transgender women may face a double jeopardy rooted in both sexism and transphobia: a dynamic that magnifies risk and normalizes mistreatment in ways that differ from the experiences of transgender men or nonbinary employees. This is not to say that transgender men do not face their fair share of workplace harassment. Saxena et al. (2023) found that transgender men faced widespread stigma and discrimination across many domains, including employment. Their findings underscore how cultural norms surrounding masculinity can uniquely shape harassment that transgender men also experience. These studies reinforce the importance of adopting an intersectional lens to the study of transgender workplace harassment, as not all transgender workers experience harassment the same way. Although individual personality traits and gender role attitudes explain who may be predisposed to harass their transgender coworkers and who is likely to be targeted for harassment, broader workplace and cultural contexts determine whether such behaviors are tolerated or discouraged. Cisheterosexism, the institutional system that privileges cisgender, heterosexual people as the norm while marginalizing those who are not, remains deeply embedded in many workplaces (Köllen & Rumens, 2022). This exclusion reflects deeper structural barriers that deny transgender employees full social and occupational legitimacy, reinforcing their vulnerability to harassment and discrimination within workplace hierarchies (Rosich, 2020). Although much research on LGBTQ+ employee experiences at work has focused on sexual minorities, transgender employees often experience a distinct form of marginalization as gender minorities, regardless of sexual orientation. Leadership plays a critical role in shaping whether harassment is tolerated or condemned. Inclusive climates and leadership (Perry et al., 2021) can signal acceptance and accountability. Conversely, supervisors who ignore complaints or model harassment themselves signal permissiveness. Visible enforcement of anti-harassment policies substantially reduces such incidents (Willness et al., 2007). Workplaces characterized by masculinity contest cultures, where success is defined by dominance, toughness, and competition, may be particularly hostile for transgender employees (Berdahl et al., 2018). In these settings, transgender employees are perceived as violating rigid gender boundaries, and harassment (e.g., misgendering, bullying, exclusion) may function to police gender norms. These dynamics can also trigger identity threats among men who fear appearing weak or tolerant of gender nonconformity, prompting them to reassert masculinity through harassment. Consistent with this, Glick et al. (2018) found that masculinity contest cultures correlate with higher levels of bullying, sexual harassment, and burnout (though these authors did not examine transgender employment specifically). Recent work in medical contexts similarly suggests that these environments may reinforce trans exclusion and harassment. For example, LGBTQ+ cardiology professionals, particularly transgender respondents, reported high rates of bias and isolation within rigidly gendered, status-oriented workplace cultures (Cook et al., 2025). Such findings align with evidence that male-dominated or gender-policing environments predict greater transphobia (Ozturk & Tatli, 2016). Workplace cultures of cisnormativity that assume everyone is cisgender (e.g., binary dress codes, gendered restrooms, pronoun neglect) may create ambient stigma for transgender employees (Brewster et al., 2012). Such cultures may contribute to an oversight in overall workplace policies, leaving transgender people especially unprotected from instances of harassment, showing that this problem is not limited to male-dominated environments in which masculinity contest culture is the norm. Both Irwin (2002) and Suárez et al. (2022) found that discrimination against transgender employees persisted even in educational settings, a field that is often publicly associated with diversity and equity. Such findings suggest that cisheteronormative norms can quietly shape institutional culture across sectors, perpetuating harassment despite formal inclusionary commitments. Having explicit workplace policies, like having a written non-discrimination policy that includes gender identity or offering trans-inclusive healthcare benefits in the workplace, can considerably improve the workplace climate for transgender people (and LGBTQ+ people in general) and contribute to higher rated job quality and a decrease in experienced harassment (Baiocco et al., 2023; Witte et al., 2020; Yu & Lee, 2024). It is important, however, to not only have these policies but to enforce them, as prior research has found organizations that showcase their values and policies without enforcing them garner more negative evaluations and lower job satisfaction Ewton and Lingas (2015). Consistent with this, Sears et al. (2024) found that LGBTQ employees working in organizations with explicit and actively enforced non-discrimination policies reported significantly lower rates of workplace harassment and greater perceptions of fairness and safety. Their findings highlight the importance of organizational accountability and policy implementation in shaping inclusive workplace climates.

Finally, larger cultural norms can present extreme challenges to prevent transgender harassment. Religious beliefs and strict gender roles in Asian Islamic cultures for instance, treat transgender people as immoral and having psychological disorders (Mamun et al., 2016; Yousuf et al., 2022). In some countries, even if anti-harassment laws exist, they may not protect trans workers (Shankar, 2024). Thus, while research on transgender harassment in non-Western cultures is currently underrepresented, we recognize the challenge in conducting research in culturally conservative locations.

### 3.3. Harassment’s Impact on Employee Well-Being

Transgender employees continue to experience disproportionately high levels of workplace harassment, discrimination, and marginalization, and poorer mental health outcomes than their non-trans peers (Cancela et al., 2025; Hill et al., 2023; Oliveira et al., 2025). And because transgender people, who are fifteen times more likely to be unemployed than the rest of the population in the United States, are not only more likely to struggle with getting employed, but also remaining employed (Ciprikis et al., 2020; James et al., 2016), it is important to understand how harassment in the workplace further contributes to such outcomes. In addition, not only are transgender employees more likely to face harassment compared with other groups, the negative impacts of harassment may be larger for transgender employees than others (Perales et al., 2025).

Table 3 summarizes 14 studies with relevance to transgender people’s harassment and mental health outcomes. While several studies show high rates of harassment among trans populations and poorer mental health outcomes for trans employees relative to their peers, research directly and causally connecting workplace harassment to trans people’s mental health outcomes is lacking. In fact, we found no study that causally tested the link between workplace harassment and mental health outcomes. Thus, at present, evidence linking workplace harassment to employee well-being is suggestive at best, relying mostly on cross-sectional correlational studies. Nonetheless, based on the available evidence, the indirect picture paints a bleak picture for transgender people’s mental health and well-being because of workplace harassment experiences.

A recent study by Cancela et al. (2024) is informative. The authors conducted a longitudinal study of 130 trans participants and found that experiences of workplace microaggressions at the beginning of the study predicted emotional exhaustion three months later. While not strictly a causal test, the time sequence suggests a causal relationship from harassment to exhaustion.

Research in STEM fields by Cech and Waidzunas (2021) found that LGBTQ+ employees, including transgender individuals, reported substantially higher frequencies of stress, insomnia, depressive symptoms, and general health difficulties compared to their non-LGBTQ+ peers. These outcomes were mediated by career limitations, devaluation, and marginalization—structural experiences that made trans workers more likely to consider leaving both their positions and their professions entirely. Although not directly assessing harassment, their findings emphasize that exclusionary work environments are not merely uncomfortable; they are materially harmful to trans employees’ mental and emotional well-being.

Analyses from a large Australian workplace survey (Perales et al., 2025) found large negative relationships between sexual harassment and employee well-being, and the relationships were strong for LGBTQ+ employees compared with non-LGBTQ+ employees, though the survey did not distinguish transgender people from other LGBTQ+ groups. Similarly, a Canadian cross-sectional study of LGBTQ workers found that compared with cisgender men, trans workers reported poorer mental health (Owens et al., 2022). Although the authors did not ask about harassment experiences specifically, trans workers also reported feeling more unsure or negative about their work environment and felt less supported; Trans workers reported using substances to cope with work stressors at higher rates than their cis male counterparts.

Workplace victimization has been linked to increased risk for hazardous substance use as well. Hinds et al. (2024) found that sexual harassment and assault predicted hazardous alcohol and drug use among sexual and gender minority (SGM) individuals. Importantly, it was victimization, rather than general discrimination alone, that most strongly predicted these outcomes, suggesting the need for interventions that address both overt acts of violence and the broader climates that enable them. These findings are reinforced by research on minority stress and psychological distress. Watson et al. (2019) demonstrated that higher levels of transgender-related harassment (not necessarily specific to the workplace) were significantly correlated with internalized transphobia, nondisclosure, and psychological distress, supporting the theoretical link between minority stress and deteriorating mental health. Similarly, Moradi (2009) found that harassment based on sexual orientation (this sample did not include transgender individuals) negatively impacted social cohesion in the workplace, which in turn reduced feelings of belonging and task-related cooperation, both of which are critical to mental wellness at work. Transgender individuals also experience elevated rates of suicidal ideation and suicide attempts across professions. Witte et al. (2020) found that transgender and nonbinary individuals reported markedly higher lifetime rates of suicidality compared with their cisgender peers in veterinary medicine, and a perceived negative work climate was associated with psychological distress, though the study did not ask about harassment experiences specifically. This suggests that while improved climate may mitigate some risk, the cumulative impact of systemic marginalization remains profound. Beyond individual mental health correlates, there are also associations between harassment and work attitudes and behaviors. In a study of Irish trans workers, 14% reported workplace harassment and 9% left a job due to harassment or discrimination, with no other job to go to (McNeil et al., 2013). Jongbloed et al. (2025) surveyed LGBTQ+ medical residents in otolaryngology, many of whom identified as transgender. They reported lower satisfaction with their residency programs and were more likely to consider leaving due to hostile or unsupportive environments. Among a U.S. sample of LGBT military vets, harassment based on sexual orientation was negatively associated with social cohesion in the workplace, which in turn reduced feelings of belonging and task-related cooperation (Moradi, 2009). Cancela et al. (2025) recently reviewed literature connecting minority stress among transgender populations, including stress experienced from workplace discrimination and harassment, and mental health outcomes. Although most studies reviewed did not directly link workplace experiences of harassment to well-being outcomes, they note that stressors experienced by trans populations are linked to a host of negative job attitudes including lower job satisfaction, lower organizational commitment, greater turnover intentions, more job anxiety, and less work engagement. Thus, workplace harassment may not only affect employee well-being, but likely damages organizational cohesion and productivity as well. Structural exclusion further exacerbates mental health disparities. Ciprikis et al. (2020) argue that the absence of explicit employment protections for transgender individuals in U.S. labor law contributes to poorer labor market outcomes and by extension, worsens mental health. The link between economic precarity and psychological distress is well-established, and when combined with stigmatization, it can produce deeply entrenched disadvantages. Oliveira et al. (2025) similarly found that LGBTQIA+ individuals, particularly transgender people, reported significantly poorer occupational health indicators and elevated absenteeism. As occupational health covers a wide variety of domains within the workplace, this suggests that LGBTQIA+ employees experience greater psychological demands, lower social support, poorer quality of relationships at work, higher rates of job insecurity, lower compensation, lower recognition at work, higher value conflicts, and lower organizational justice. This also extends to overall health and well-being, covering physical aspects such as fatigue and musculoskeletal pain, along with mental health aspects like depression and stress.

Overall, more research is needed directly linking transgender people’s work experiences (and in particular, harassment experiences) to their well-being, mental and physical health, and work attitudes and behaviors. With LGBTQIA+ employees disproportionately experiencing these negative psychosocial, health, and well-being outcomes, this may lead to greater levels of burnout and commitment to the workplace, further contributing to higher turnover and less diversity within organizations and the aforementioned unemployment rate specifically for transgender people. Organizations also likely suffer when workers are less satisfied and productive and leave their jobs.

### 3.4. Strategies to Improve Workplace Climate

Taken together, this body of research highlights a pressing need for systemic interventions aimed at dismantling workplace discrimination and fostering affirming environments for transgender workers. Addressing these challenges will require a combination of policy enforcement, cultural change, and the prioritization of mental health resources tailored to the unique needs of transgender employees.

Despite growing awareness of transgender rights in broader society, workplace environments remain inconsistent in offering meaningful protection, support, and inclusion for transgender employees. While many companies have implemented formal anti-discrimination policies, these can often fall short in their execution, leaving transgender workers vulnerable to both overt and covert forms of marginalization in the workplace. A growing body of research provides insight into the structural and cultural reforms necessary to cultivate truly inclusive and affirming work environments to decrease the harassment and discrimination that transgender employees often face in the course of their employment. This research is summarized in Table 4.

Balakrishnan and Mohaptra (2022) found that although 61% of surveyed workplaces had anti-discrimination policies on record, only 34% of non-cis participants indicated their organizations actively conducted sensitization programs about those policies. A majority of transgender respondents reported experiencing either conscious or unconscious bias at work—manifesting through limited advancement opportunities, biased performance reviews, and social exclusion. These findings underscore the distinction between having policies and implementing them effectively, echoing concerns raised by Bradley et al. (2024), who argue that signaling inclusion without operational follow-through fails to protect marginalized employees.

Addressing this gap requires organizations to move from performative declarations to active, structural support. Ewton and Lingas (2015) advocate for what they term “activating signals”. In other words, a company’s practice should focus not only on explicitly stating their commitment to diversity and inclusion, but embodying it through daily, visible actions. These signals are more effective than “pointing signals,” which merely gesture toward inclusivity without modifying institutional behaviors. For transgender employees, activating signals can include the use of gender-affirming language, accessible and gender-neutral facilities, and equitable mobility and leave policies. This is especially important as oftentimes LGBTQ+ employees have to weigh the personal costs of workplace culture. Zindel and de Vries (2024) found that many LGBTQ+ employees are willing to sacrifice aspects of their career advancement or monetary compensation for a work environment that explicitly values inclusivity and safety, highlighting just how deeply organizational culture not only shapes experiences of harassment and discrimination for transgender employees, but also broader career trajectories and employee retention.

Several studies highlight the critical role of workplace climate in shaping transgender employees’ psychological safety and job satisfaction. Fletcher and Marvell (2023) found that a positive diversity and inclusion climate moderates the impact of social dominance orientation among cisgender employees, thereby increasing allyship intentions. These outcomes were bolstered when HR practices were inclusive of a broad range of gender identities and expressions, and when justice and belonging were embedded into promotion and recruitment structures.

The importance of interpersonal support cannot be overstated. Huffman et al. (2021) emphasize that support from supervisors and coworkers—through behaviors such as respecting pronouns and discouraging derogatory language—has a more direct impact on job satisfaction and gender identity openness than organizational policies alone. Similarly, Schönauer et al. (2025) found that support from immediate colleagues and supervisors was more strongly linked to authenticity at work than were formal institutional practices, highlighting the importance of daily interactions in creating inclusive climates.

To ensure that policies translate into practice, targeted training for human resources personnel and leadership is essential. The PRIDE intervention developed by Matsutaka et al. (2024) demonstrated that structured, multi-session training for HR and health professionals significantly reduced transphobic and homophobic attitudes. Crucially, this program combined knowledge acquisition with skill-building and self-reflection, suggesting that empathy and efficacy can be taught and reinforced.

Trans-specific allyship training offers another promising avenue for decreasing workplace harassment. A recent review (Ho et al., 2023) found that LGBT allyship was generally associated with perceived support and psychological well-being. Similarly, Fletcher and Marvell (2023) surveyed trans employees and found that perceived allyship was associated with psychological safety and authenticity at work, and another study focused on the experiences of LGBTQ+ employees found that diversity training and ally networks are associated with increased well-being (Perales, 2022). That being said, evidence linking ally programs to actual reductions in harassment incidents is lacking, offering a fruitful direction for future research.

In addition to training, leadership visibility and organizational accountability are key. McQuillan et al. (2024) found that trans educators working in schools with aligned policy, organizational support, and affirming leadership reported the highest levels of safety and job satisfaction. Importantly, policy protections alone were insufficient unless accompanied by consistent leadership practices and organizational norms that disrupted cisnormativity.

Finally, linguistic inclusion plays a vital role in shaping perceptions of belonging. Perales et al. (2022) provide robust empirical evidence that exposure to inclusive language in the workplace—regardless of whether it was embedded in formal policy or day-to-day communication—was significantly associated with improved well-being and feelings of inclusion among trans employees. These findings suggest that everyday language can act as a powerful tool for social integration.

This body of research points to a multi-level approach to transforming the workplace climate for transgender employees. It is not enough to implement surface-level diversity policies; organizations must actively foster inclusion through policy enforcement, ongoing education, leadership modeling, and daily interpersonal practices. Such integrated efforts not only mitigate harassment and discrimination but also cultivate workplaces where transgender employees can thrive safely and authentically.

### 3.5. A Word on Race/Ethnicity

In our search of the literature, we were mindful of intersectionality. We looked for research that examined the workplace harassment of transgender people through and intersectional lens, by for instance, separating harassment experiences of racially or ethnically minoritized trans workers from White workers. We were unable to find any such studies. This is in obvious gap in the research today that warrants further investigation.

## 4. Discussion and Future Research

This review underscores several critical gaps in the current literature and provides a roadmap for future research focusing on the harassment that transgender employees face. While the field has made strides in recognizing the prevalence and the consequences of such harassment, the existing scholarship remains limited in scope and may, at times, reinforce normative assumptions, further perpetuating the cisheteronormativity that often contributes to such harassment in the first place. Under the framing of role congruity theory (Eagly & Karau, 2002), this review shows how those who deviate from gendered expectation can be targeted for punishment as a way of enforcing gender norms that spring from cisheteronormative assumptions, but also how these assumptions can render some forms of harassment invisible. Firstly, psychology has historically contributed to our understanding of prejudice, stereotyping, and discrimination through frameworks that often center the experiences of cisgender, heterosexual individuals. Consequently, transgender people are frequently subsumed within broader LGBTQ+ samples, which may not account for unique experiences that are specifically tied to their gender identities. For example, dead-naming and bathroom exclusion policies are forms of workplace harassment unique to transgender populations. Moreover, existing research as well as real-world workplace policies and practices often reinforce binary views of gender that render gender non-conforming and non-binary people invisible. Future research must challenge the tendency to group transgender people together. Transgender people are not a monolith, and future research must recognize that the experiences of transgender men, transgender women, and others beneath the transgender umbrella (e.g., non-binary people) are not shaped only by their shared transgender identity. For example, transgender women may experience harassment that is rooted in transmisogyny, while non-binary individuals may encounter additional harassment that is based in their invalidation due to expectations tied to traditional gender roles. Future research should adopt intersectional and identity-contingent approaches to examine how gender identity interacts with other social categories (e.g., race, age, perceived gender conformity) to shape workplace experiences.

Second, researchers should expand the cultural lens through which they study transphobic workplace harassment. Much of the current literature is based in Western contexts, which limits the generalizability and can obscure how local cultural, governmental, and organizational factors shape transgender employees’ experiences in other areas of the world. Comparative research across cultural contexts can help to illuminate how cisnormativity functions differently in various workplace settings and how different governmental and cultural frameworks enable or constrain some of the inclusive practices mentioned in this review. Third, current models of workplace harassment often fail to account for the contextual and structural dimensions of bias, particularly cisnormativity. Many studies in this review focus on individual-level predictors or outcomes without sufficiently analyzing the systemic or structural conditions that enable or encourage transphobic harassment within the workplace. Future research must take the role of cisnormativity seriously as a dominant ideology that not only marginalizes transgender people in general, but also legitimizes and, at times, obscures the harassment and harm they face. An understanding of how workplace norms–particularly those grounded in masculinity contest cultures and binary gender ideologies–shape the form and frequency of harassment is essential in decreasing and, ultimately, overcoming this issue in the workplace. Researchers should explore how cisnormativity not only functions as a background assumption, but as an active force in the production and maintenance of work environments that are hostile toward transgender people. Therefore, future research would benefit from integrating models of workplace harassment that emphasize the relationship between individual attitudes, group norms, organizational structures, and cultural ideologies. Fourth, despite the clear evidence that transgender people face widespread harassment, the ways in which researchers conceptualize and operationalize gender-based harassment draws on definitions that were originally developed with cisgender women in mind. This review underscores the importance of interrogating how these definitions should evolve to reflect the realities of transgender employees and transgender people in general. Future research should include a critical examination of how gender-based harassment is defined, measured, and interpreted, especially when accounting for both explicit and implicit forms of transphobic harassment in the workplace (e.g., deadnaming, misgendering, exclusion from gendered spaces, and performative actions to mask deeper, structural hostility). Lastly, researchers must prioritize community-engaged methods that center transgender voices: not only as research subjects, but active collaborators in the production of knowledge surrounding experiences of harassment in the workplace. Transgender-led research and collaboration with community organizations can ensure that research remains grounded in lived experiences and is oriented toward real, transformative change rather than performative obstruction.

## 5. Conclusions

Workplace harassment of transgender people is quite prevalent, even normative, as a tool to reinforce gender role conformity (Eagly & Karau, 2002). Harassment may be particularly likely in environments that valorize masculinity. Harassment may be underestimated by organizations, because some forms of harassment are unique to trans populations and do not fit traditional definitions of harassment, leaving trans populations more vulnerable and less protected than other populations. This is reflected in the myriad of poor mental health outcomes and organizational inefficiencies that result from harassment. Looking ahead, organizations must move beyond performative inclusion and incorporate more nuance and context-rich understandings of the experiences trans people have in the workplace. Laws, organizational policies, and employee training should be tailored to recognize transgender workers’ rights and protections to dismantle the hostile environments that continue to contribute to workplace harassment and ultimately harm transgender employees, perpetuating systemic inequalities both in and outside of the organization. At the same time, social and organizational psychologists must work to fully integrate transgender experiences into existing theoretical frameworks on workplace behavior and harassment. Much of the research on LGBTQ+ employees has, in practice, centered on sexual minorities, leaving gender minorities comparatively invisible. More is known about the predictors and outcomes of harassment for lesbian, gay, and bisexual employees than for transgender and nonbinary employees. Expanding psychological theory to explicitly include gender identity beyond sexual orientation will not only fill in critical gaps in understanding, but also improve the precision and inclusivity of research on improving outcomes in the workplace for transgender employees. Doing so will not only ensure that psychology contributes meaningfully to creating workplaces that recognize, protect, and affirm transgender employees, but will also enhance broader efforts to improve the occupational health, well-being, and the everyday lived experiences of transgender people who continue to face disproportionate harassment and marginalization across many domains.

## Figures and Tables

**Table 1:** Studies reporting the prevalence of transgender workplace harassment.

Study	Description
Barthelemy et al. (2022)	37 trans academic physicists, mostly from the U.S. 49% report exclusionary behavior; 3035% report uncomfortable climate.
Casey et al. (2019)	U.S. sample (N = 86). 38% experienced slurs, 28% micoaggressions (not specific to work). 51% experienced sexual harassment (not specific to work; percentages are for all lgbt group members, not transgender specific).
Cech and Waidzunas (2021)	U.S. STEM professional (N = 1006 LGBTQ, but did not separate trans); roughly 20% experienced harassment in past year
Chester et al. (2014)	S. sample of 42 LGBT (unknown number of trans) medical students, residents, physicians. 12% experienced harassment at their medical center
Cook et al. (2025)	U.S. sample of cardiology physicians and fellows in training (62 LGBTQ; 7 non-cis identifying). Didn’t separate trans from LGBTQ. 75% experienced ‘negative experiences’ (e.g., exclusion, gossip/rumors, hostility) in past year
Enogieru et al. (2024)	U.S. sample (N = 4597). 2008–2009 National Transgender Discrimination Survey; 50% had experience harassment at work.
European Union Agency for Fundamental Rights (2024)	N = 100,000+ LGBTQI+ people from the EU and neighboring countries. A majority of trans people in every country queried report being harassed in the past 12 months, but this was not specific to the workplace
James et al. (2016)	U.S. sample (N = 27,715). 15% verbally harassed, physically attacked, and/or sexually assaulted at work in the past year; 23% other forms: being told by their employer to present as the wrong gender in order to keep their job or having employers or coworkers share private information about their transgender status with others without permission.
Medina and Mahowald (2023)	U.S. sample (N = 1828 LGBTQI+); for trans respondents: 51% verbal, 41% sexual, 21% physical harassment at work in past year. 26% excluded from work events; 23% denied access to restrooms. 70% experienced some form of harassment.
Prunas et al. (2018)	Italian sample (N = 68); 22% reported harassment in the workplace.
Robinson et al. (2024)	Australian national online survey of 1000 LGBTQ aged 14–30 (trans people not separated from other LGB groups). 77% experienced workplace sexual harassment.
Schuyler et al. (2020)	U.S. military (N = 56 gender minority); 83.9% experienced sexual harassment.
Sears et al. (2024)	U.S. sample (N = 84 transgender or nonbinary participants). 68% had experienced at least one form of workplace harassment (verbal, physical, sexual) during careers; 56% in the past five years.
Suen et al. (2021)	10,000+ Chinese LGBTI workers (691 trans people). 31.4% reported “negative treatment” (higher than the 21.2% overall among LGBTI).
Waite (2021)	235 Canadian Federal Public service workers. 31.27% reported harassment in past 2 years.

**Table 2:** Examples of forms of workplace harassment of transgender people.

Study	Description
Baiocco et al. (2023)	Developed a Job Quality Index for LGBTQ+ people that included items measuring supportive and hostile work environments (e.g., invasive, indiscreet questions, transphobic comments, deadnaming, misgendering, making you feel it would be preferable if you were cisgender).
Brewster et al. (2014)	Surveyed American trans workers. A workplace experiences questionnaire included items about being ignored, made crude or offensive sexual remarks, made transphobic remarks, asked personal questions, offensive jokes.
Cancela et al. (2024)	A 3-month longitudinal study of workplace microaggressions experienced by transgender people. Respondents reported a variety of behaviors including deadnaming, misgendering, transphobic slurs, invasions of privacy and invasive questioning, being stared at, gossiped about, and being outed.
Galupo and Resnick (2016)	Reports a survey of microaggressions experienced by LGBT workers (13% trans). They asked respondents about three categories of microaggressions: microassaults (verbal and nonverbal attacks), microinsults (rudeness or insensitivity), and microinvalidations (communications that negate or nullify psychological thoughts, feelings, or experiential reality).
Herman (2013)	Surveyed transgender and gender nonconforming people about their experiences in gendered public restrooms. The majority reported being denied access, verbally harassed, or physically assaulted in public restrooms.
Perales et al. (2022)	“Examples of sexual harassment include being the target of unwelcome/inappropriate physical contact, sexually explicit comments or gestures, receiving intrusive questions about your private life, and inappropriate advances or requests for sex.”
Resnick and Galupo (2019)	Developed a “LGBT Microaggressions Experiences at Work Scale.” Some of the items described harassing behaviors unique to transgender workers (e.g., hearing a colleague or customer being called a “tranny”; being “tokenized”; deadnaming; misgendering; bathroom exclusion; clothing enforcement).
Van de Cauter et al. (2021)	A review of research on return to work of transgender employees following medical leaves. Trans workers reported frequent deadnaming, misgendering, and asking inappropriate questions. Some reported name-calling, being reported for restroom use, and social exclusion.
Waite (2021)	Surveyed Canada’s federal public service workers. Defined harassment as “ objectionable act(s), comment(s) or display(s) that demean, belittle, or cause personal humiliation or embarrassment, and any act of intimidation or threat.
Watson et al. (2019)	Reports the development of the Trans Discrimination Scale, which includes items about harassment and microaggressions (e.g., refused to use your gender pronouns, received demeaning messages about your appearance, heard intrusive comments about your body)

**Table 3:** Studies linking harassment to employee well-being.

Study	Description
Cancela et al. (2024)	A longitudinal study of workplace microaggressions experienced by transgender people found that harassment at Time 1 predicted emotional exhaustion 3 months later.
Cancela et al. (2025)	This review paper links trans minority stress to a host of negative job attitudes and mental health outcomes.
Cech and Waidzunas (2021)	LGBTQ+ STEM employees, including transgender individuals, reported substantially higher frequencies of stress, insomnia, depressive symptoms, and general health difficulties compared to their non-LGBTQ+ peers.
Ciprikis et al. (2020)	Reports analyses of U.S. wage gap and employment gap data comparing trans and non-trans workers. They suggest that “43 per cent of the wage differential is unexplained and may be due to discrimination.”
Hill et al. (2023)	Australian study (1446 trans people). Suicide attempts were higher among those who experienced sexual harassment; Suicide ideation higher for those who felt less accepted at work.
Hinds et al. (2024)	Sexual harassment and assault predicted hazardous alcohol and drug use among a sample U.S. sexual and gender minority (SGM) individuals; harassment experiences were not specific to the workplace.
James et al. (2016)	Analyzed 2015 U.S. transgender survey. 77% who had a job in the past year took steps to avoid mistreatment in the workplace, such as hiding or delaying their gender transition or quitting their job.
Jongbloed et al. (2025)	LGBTQ+ medical residents in otolaryngology, many of whom identified as transgender, reported lower satisfaction with their residency programs and were more likely to consider leaving due to hostile or unsupportive environments.
Moradi (2009)	Among a U.S. sample of LGBT military vets (N = 445), harassment based on sexual orientation was negatively associated with social cohesion in the workplace, which in turn reduced feelings of belonging and task-related cooperation.
McNeil et al. (2013)	Irish study (N = 164 trans participants). 14% reported workplace harassment. 9% left a job due to harassment or discrimination, with no other job to go to
Oliveira et al. (2025)	Portuguese sample. LGBTQIA+ individuals, particularly transgender people, reported significantly poorer occupational health indicators and elevated absenteeism, but the study did not ask about harassment.
Perales et al. (2025)	Data from large (45,000) Australian workplace survey; found large negative relationships of sexual harassment and employee well-being, and the relationships were strong for LGBTQ+ employees. Did not separate trans from other LGBTQ+ populations.
Watson et al. (2019)	As part of the development of a trans discrimination scale, higher levels of transgender-related harassment were significantly correlated with internalized transphobia, nondisclosure, and psychological distress.
Witte et al. (2020)	Transgender and nonbinary individuals reported markedly higher lifetime rates of suicidality compared with their cisgender peers in veterinary medicine.

**Table 4:** Studies with implications for policies and practices to improve workplace environments for transgender employees.

Study	Description
Balakrishnan and Mohaptra (2022)	Highlights the difference between having formal policies and actively enforcing the policies. Although 61% of surveyed workplaces had anti-discrimination policies on record, only 34% of non-cis participants indicated their organizations actively conducted sensitization programs about those policies.
Bradley et al. (2024)	Signaling inclusion without operational follow-through fails to protect marginalized employees.
Ewton and Lingas (2015)	Advocate for “activating signals”—embodying commitment to diversity through everyday, visible actions.
Fletcher and Marvell (2023)	Discusses allyship. Positive diversity and inclusion climate moderates the impact of social dominance orientation among cisgender employees, thereby increasing allyship intentions. Among transgender employees, perceived allyship was associated with greater psychological safety, authenticity, and life satisfaction.
Ho et al. (2023)	A scoping review of the importance of allyship for LGBTQ+ employees’ well-being.
Huffman et al. (2021)	Support from supervisors and coworkers has a more direct impact on job satisfaction and gender identity openness than organizational policies alone.
Matsutaka et al. (2024)	Describes the PRIDE training intervention for human resources professionals to reduce workplace transphobia and homophobia.
McQuillan et al. (2024)	Trans K-12 educators working in schools with aligned policy, organizational support, and affirming leadership reported the highest levels of safety and job satisfaction.
Perales (2022)	Diversity training and ally networks are associated with increased well-being among LGBTQ+ employees.
Perales et al. (2022)	Linguistic inclusion, formal or informal, significantly associated with improved well-being and feelings of inclusion among trans employees.
Schönauer et al. (2025)	Support from immediate colleagues and supervisors was more strongly linked to authenticity at work than were formal institutional practices
Zindel and de Vries (2024)	Reports and experiment showing that many LGBTQ+ employees are willing to sacrifice aspects of their career advancement or monetary compensation for an inclusive, safe work climate.

## Data Availability

No new data were created or analyzed in this study.
